# Social determinants of health and diabetes: using a nationally representative sample to determine which social determinant of health model best predicts diabetes risk

**DOI:** 10.1186/s40842-023-00162-5

**Published:** 2024-02-25

**Authors:** Zach W. Cooper, Orion Mowbray, Leslie Johnson

**Affiliations:** 1 University of Georgia School of Social Work, 279 Williams Street, Athens, GA 30602 Georgia; 2 Department of Family and Preventative Medicine, School of Medicine, Emory University, Atlanta, Georgia

**Keywords:** Social determinants of health, Diabetes, BMI, Diabetes disparities

## Abstract

**Objectives:**

Social determinants of health (SDOH) research demonstrates poverty, access to healthcare, discrimination, and environmental factors influence health outcomes. Several models are commonly used to assess SDOH, yet there is limited understanding of how these models differ regarding their ability to predict the influence of social determinants on diabetes risk. This study compares the utility of four SDOH models for predicting diabetes disparities.

**Study design:**

We utilized The National Longitudinal Study of Adolescent to Adulthood (Add Health) to compare SDOH models and their ability to predict risk of diabetes and obesity.

**Methods:**

Previous literature has identified the World Health Organization (WHO), Healthy People, County Health Rankings, and Kaiser Family Foundation as the conventional SDOH models. We used these models to operationalize SDOH using the Add Health dataset. Add Health data were used to perform logistic regressions for HbA1c and linear regressions for body mass index (BMI).

**Results:**

The Kaiser model accounted for the largest proportion of variance (19%) in BMI. Race/ethnicity was a consistent factor predicting BMI across models. Regarding HbA1c, the Kaiser model also accounted for the largest proportion of variance (17%). Race/ethnicity and wealth was a consistent factor predicting HbA1c across models.

**Conclusion:**

Policy and practice interventions should consider these factors when screening for and addressing the effects of SDOH on diabetes risk. Specific SDOH models can be constructed for diabetes based on which determinants have the largest predictive value.

## Background

Diabetes is one of the most common chronic diseases in the United States, affecting 11.3% of the population annually [1]. Diabetes generates an estimated $327 billion dollars in annual medical costs, with an average of $9600 in excess healthcare costs per person [2]. Diabetes is also one of the leading causes of death, and annual deaths are expected to increase from 146,604 to 385,800 between 2015 and 2030 in the United States [3–5].

Diabetes has complex biological and psychosocial mechanisms of influence [6, 7]. Despite this, the history of diabetes treatment has emphasized genetics and health behaviors, largely neglecting social and environmental factors influencing diabetes risk [8].

Recently, social determinants of health (SDOH) have emerged as a strategy for addressing health disparities [9]. Prior research has demonstrated the influence of social determinants on the development of common chronic conditions such as cardiovascular disease [10], sleep disorders [11], cancer [12], and diabetes [9, 13]. These findings expand the more reductionistic model of chronic disease development which emphasizes genetics and health behaviors [14].

An increasing amount of research has examined how SDOH influence diabetes risk, with four prevailing models being employed. A recent systematic review summarizing the literature on SDOH and diabetes outcomes references the World Health Organization (WHO), Healthy People, County Health Rankings, and the Kaiser Family Foundation as the primary theoretical models summarizing SDOH [9]. These models categorize SDOH as economic/social context, social support, material circumstances, and health care status [9]. Each SDOH model operationalizes these categories distinctly which has important implications for the construction of statistical models. For example, The Healthy People model uses 1) civic participation, discrimination, and social cohesion to define the social and community context [9], and the County Health Rankings Model uses 1) family and social support and 2) community safety to operationalize the community context [15]. Since each SDOH model emphasizes different SDOH factors, understanding which model is most predictive of diabetes risk is pivotal for addressing disparities in diabetes research.

Little research has been conducted to compare the function of SDOH models for examining health disparities. Much of the existing literature isolates singular constructs included within SDOH models to include income [16], housing [17], environmental exposure [18], and social support [19]. The purpose of the current study is to 1) operationalize SDOH models utilizing The National Longitudinal Study of Adolescents to Adult Health (Add Health) dataset, 2) utilize mixed regression modeling to determine which SDOH model best predicts diabetes risk, and 3) identify which SDOH factors are the strongest predictors within each model. The current approach seeks to build on the existing literature utilizing general systems theory and the person in environment perspective from which the SDOH models were influenced [20].

## Method

### Study design and participants

This study included participants from a nationally representative longitudinal study with over 20,0000 participants which assesses health and mental health development from adolescence into adulthood [21]. The initial Add Health data were collected in 1994–1995 when participants were in the 7th–12th grade. Researchers utilized a stratified sample of 80 high schools, each school had at least 30 students. Schools were stratified by size, race/ethnicity, school type, urbanicity, and geography.

The in-home survey for the Wave I data included 20,745 participants. Subsequent waves included 14,738 participants for Wave II, 15,197 for Wave III, 15,701 for Wave IV, and 12,300 for Wave V. Biological data were also collected in Wave V, with HbA1c data reported. The current analyses utilize data from the Wave V in-home survey and the biomarker data for HbA1c. Data were collected between 2016 and 2018 and there was a total of 11,985 participants who completed the survey in its entirety. The data for HbA1c was collected from 2016 to 2019. The labs were conducted by registered phlebotomists who were all trained on the Add Health protocol. There was a total of 1631 participants who had a HbA1c reading. The Add Health data set was selected due to its national representation and inclusion of comprehensive health variables to include HbA1C and BMI. This study was approved by the Intuitional Review Board of the University of Georgia.

### Measurement

The Kaiser, WHO, Healthy Peoples, and County Health Rankings models are four of the most used SDOH models [9]. Researchers were interested in testing these four SDOH models to analyze their efficacy in predicting diabetes risk. As a result, these four models were used to operationalize the SDOH variables included within each statistical model, see Table 1. The Add Health dataset was utilized to operationalize variables related to demographics, social determinants, and diabetes outcomes. Researchers used the Add Health to build each of the statistical models included in our analyses. Table 1 provides an overview of how the SDOH models are operationalized and Tables 3 and 4 lists each of the variables used from the Add Health dataset to create each of the SDOH models.

**Table 1:** SDOH Models

SDOH Model	Socioeconomic Factors	Education and Social Class	Social and Community Factors	Physical Environment	Health Care	Food Environment
World Health Organization	**Socioeconomic and Political Context** -Governance -Macroeconomic Policies -Social and Public Policies -Culture and Societal Values	**Socioeconomic Position** -Social Class -Gender -Ethnicity (racism) -Education -Occupation -Income	**Social Cohesion and Social Capital**	**Material Circumstances** -Housing and neighborhood quality -Basic Needs -Physical Work environment	**Health Care** -Health Access	None
Healthy People	**Economic Stability** -Employment -Food insecurity -Housing instability -Poverty	**Education** -Early childhood education -Higher Education -High school graduation -Language development and literacy	**Social and Community Context** -Civic participation -Discrimination -Incarceration -Social cohesion	**Neighborhood and Built Environment** -Access to healthy food -Community safety -Environmental conditions -Housing Quality	**Health and Health Care** -Access to health care -Access to primary care -Health Literacy	None
County Health Rankings Model	**Economic Factors** -Education -Employment -Income	None	**Social Factors** -Family and social support -Community safety	**Physical Environment** -Air and water quality -Housing	None	None
Kaiser Family Foundation	**Economic Stability** -Employment -Income -Expenses -Debt -Medical bills -Support	**Education** -Literacy -Language -Early Childhood Education -Higher Education	**Community and Social Context** -Social integration -Support systems -Discrimination -Community Engagement -Stress	**Neighborhood and Physical Environment** -Housing -Transportation -Safety -Parks and Playgrounds -Walkability -Zip Code	**Health System** -Health Coverage -Provider Availability -Provider cultural competency -Healthcare Quality	**Food Environment** **-**Hunger -Access to healthy food

Hill-Briggs, F. et al. Social Determinants of Health and Diabetes: A Scientific Review. *Diabetes Care* 44, 258–279 (2021)

#### Demographic

There were several demographic questions which participants were asked to respond to including age (in years), gender (female vs. male), race, and household income. Participants were asked to self-identify their race/ethnicity from the following eight response options: White, Black/African American, Hispanic, Asian, Pacific Islander, American Indian/Alaskan Native, Other, and Multiracial. These demographics were dichotomized with Non-Hispanic White participants operating as the reference group. Income included 13 different options with income ranging from “less than $5,000” to “$200,000” or more. Though the Add Health dataset did report household income, they did not report household income in proportion to the number of individuals included within the household. The results regarding the influence of household income should be interpreted with that limitation.

#### HbA1c

HbA1c is considered one of the most important biomarkers used to assess diabetes risk and to inform diabetes treatment [22]. HbA1c provides an average of the patient’s blood glucose levels over the past 3 months and is considered a standard of diabetes assessment and treatment [23]. Within the Add Health dataset there were 1426 participants who had their HbA1c read and who completed their Wave V survey. There were issues with skewness and kurtosis with this variable. HbA1c was therefore dichotomized to those at risk for diabetes (HbA1C > 5.7%) and those who were not at risk (HbA1C < 5.7%), this is consistent with clinical guidelines for diabetes [23].

#### BMI

Obesity is highly predictive of diabetes risk and is therefore a standard to predict risk in diabetes research and clinical practice [24]. Obesity increases diabetes risk through insulin resistance and deficiency [24]. Body mass Index (BMI) is conventionally utilized to determine whether a patient is overweight. Weight and height variables were utilized to create a BMI variable within the Add Health dataset (weight*703)/(height**2). The sample frame of 1426 participants that was used for the HbA1c analysis was also used for BMI. Normality assumptions were met without any need for transformation for BMI.

#### Social determinant of health models

The four leading SDOH models were utilized to determine which model best predicted diabetes outcomes [9]. Each of the models included categories (i.e., Economic stability) with multiple variables constructing those categories (i.e., Income, Education). Each of the models are described below. See Table 1 for a detailed description of how each SDOH model was operationalized [9].

##### County health rankings

The County Health Rankings Model included three categories: economic factors, social factors, and the physical environment [15]. Constructs comprising economic factors include education, employment, and income. Social factors were comprised of family and social support and community safety. Last, the physical environment was defined by air and water quality as well as housing and transit quality. The Add Health data set had questions which asked participants whether they had individuals whom they could rely on and others which asked if they could open-up to these individuals. These variables were dichotomized, with those who indicated they had someone they could rely on or open-up to as “1” and those who did not have someone they could rely on or open-up to as “0”.

##### World Health Organization

The WHO included five major categories to define SDOH. These included socioeconomic and political context, socioeconomic position, social cohesion and social capital, material circumstances, and health care [19]. Socioeconomic and political context included macroeconomic and social policies as well as cultural and societal values. Socioeconomic position consisted of social class, gender, ethnicity, education, and income. Participant’s geographic location was used as a proxy for social policies and cultural and societal values. An electoral college map was used to designate the geographical regions as more liberal or conservative [25]. This variable was then dichotomized for more conservative regions being labeled as “1” and more liberal regions being labeled as”0.” Insurance status was used to represent access to healthcare. There are other factors such as the ability to pay out of pocket which could also influence healthcare access, but these data were not included in the Add Health dataset. Income was included within most of the SDOH models to account for this.

##### Healthy people

The Healthy People Model included 5 categories to operationalize SDOH. These included economic stability, education, social and community context, neighborhood and built environment, and health care*.* Civic engagement, discrimination, incarceration history, and social cohesion represented the social and community context category. Economic stability was comprised of food insecurity, poverty, and employment status. The Add Health dataset included questions which asked about social interaction. These variables were used as a proxy for social cohesion.

##### Kaiser Permanente

The Kaiser Permanente Model consisted of 6 categories to include economic stability, education, community and social context, neighborhood and physical environment, health and system, and food environment. Medical bills, debt, and having social support were included in the economic stability category. Stress, social support, and support systems were utilized in the construction of community and social context. The Add Health dataset included a question which asked how much total household debt participants had. This variable was dichotomized where those who had any debt as being “1” and those with no debt as being “0.” The dataset included a variable which asked the frequency in which the participant had felt overwhelmed. This variable utilized a Likert scale and was used to represent stress.

### Analysis

After merging the biomarker data including HbA1c with the survey data, there were a total of 1426 participants included within the analysis. A series of T-Tests were run at the bivariate level to assess relationships between variables. A total of eight regression analyses were run: four logistic regression analyses to analyze odds ratios for SDOH models on HbA1c and four linear regression analyses to analyze influence of SDOH models on BMI.

## Results

Demographic analyses were conducted for these 1426 participants included within the final model. Most of the sample identified as female (61.7%). Regarding race and ethnicity, 72.37% of participants identified as White, 18.45% identified as Black, and 9.18% identified as another race or ethnicity. The remaining racial groups were collapsed into an “Other” category due to low cell counts. See Table 2 for a full sample description.

**Table 2:** Sample characteristics

	Number	Percent
***Gender***		
Female	997	61.7
Male	619	38.3
***Racial Identity***		
White	1148	72.37
Black	291	18.45
Other Race	138	9.18
***Household Income***		
Less than $5000	171	10.67
$5000–$24,999	270	16.85
$25,000–$49,999	443	27.65
$50,000–$99,999	495	30.90
$100,000–$199,999	184	11.49
$200,000 or more	39	2.43
***Education***		
No High School Degree	64	4.08
High School or GED	191	12.17
Some College	424	27.02
Associate or Junior College	173	11.03
Bachelor’s degree	377	24.03
Some Graduate School	64	4.08
Completed Masters	228	14.53
Completed Doctoral	48	3.06
**Total**	1569	100

Factors within the County Health Rankings model that significantly increased the risk for elevated HbA1c included identifying as Black (OR = 3.32, *p* < .01), or as another racial minority (OR = 1.80 *p* < .05), and being in poverty (OR = 1.59, *p* < .05). Regarding BMI, the model was significant [F (12, 1413) = 7.76, *p* < .0001] and accounted for 6.2% of the variance in BMI. Unique main effects for BMI included identifying as Black (t = 5.43, *p* < .01) and poverty (t = 2.87, *p* < .01).

Factors within the WHO model that significantly increased the risk for elevated HbA1c included identifying as Black (OR = 4.08, *p* < .01) and gender (OR = 1.55, *p* < .05). Regarding BMI, the model was also significant [F (13, 1366) = 9.05, *p* < .01] and accounted for 8.9% of the variance in BMI. Unique main effects for BMI included identifying as Black (=4.93, *p* < .01) as well as social status (t = − 5.66, *p* < .01).

Factors within the Healthy People model that significantly increased the risk for elevated HbA1c included identifying as Black (OR = 3.84, *p* < .01), male (OR = 1.47, *p* < .01), history of speech and literacy challenge (OR = 2.92., *p* < .01), poverty (OR = 1.56, *p* < .01), and incarceration history (OR = .63, *p* < .05). Regarding BMI, the model was also significant [F (16, 1413) = 6.04, *p* < .01] and accounted for 7.0% of the variance in BMI. Unique main effects for BMI included identifying as being Black (t = 5.35, *p* < .01), access to higher education (t = − 2.64, *p* < .05), poverty (t = 2.38, *p* < .05) history of incarceration (t = − 2.23, *p* < .05), and lack of healthcare access (t = 2.19, *p* < .05).

**Fig. 1:**
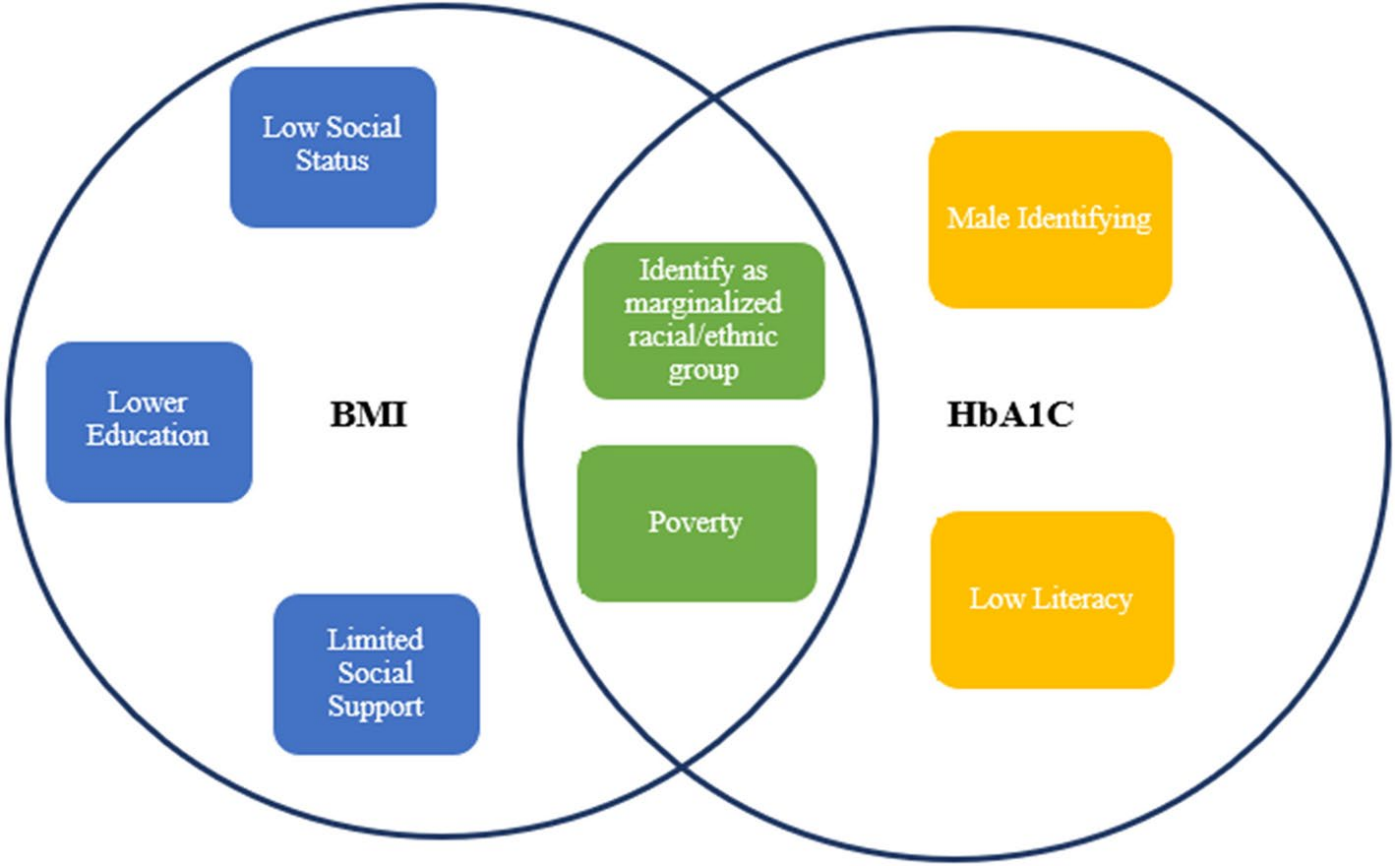
Risk factors for elevated HbA1c and high BMI

Factors within the model that significantly increased the risk for elevated HbA1c included identifying as Black (OR = 4.81, *p* < .01), literacy challenges (OR = 3.60, *p* < .01), and healthcare quality (OR = 1.81, *p* < .01). Regarding BMI, the model was also significant [F (31,1287) = 9.49, *p* < .01] and accounted for 19.0% of the variance in BMI. Unique main effects for weight included identifying as Black (t = 4.33, *p* < .01) having a spouse to rely on (t = − 2.86, *p* < .01), access to higher education (t = − 2.74, *p* < .01), debt (t = 2.46, *p* < .05), and healthcare quality (t = 12.25, *p* < .01). See Table 3 for a summary of the results regarding BMI and Table 4 for a summary of the results regarding HbA1c. See Fig. 1 for a visual depiction of the significant results for both BMI and HbA1c.

**Table 3:** Regression for BMI

Independent Variable	County Health Ranking Model (1569)		WHO Model (1569)		Healthy People Model (1569)		Kaiser Model (1569)	
	t	B	t	B	t	B	t	B
Black	5.43**	2.75						
Other Race	−2.36*	−2.58						
Gender	−0.60	−0.24						
Housing	0.34	0.23						
Employment	0.99	0.56						
Education	−1.96	− 1.51						
Rely On Spouse	−3.97	−1.96**						
Rely On Children	0.02	0.04						
Rely On Friends	−2.14	−1.21*						
Community Safety	−0.24	−0.16						
Income	2.87**	1.20						
Black			4.93**	2.58				
Other Race			−2.44*	−2.72				
Gender			0.60	0.25				
Political Context			1.12	0.49				
Social Class			−5.66**	−0.66				
Education			−1.19	−0.93				
Work Status			0.67	0.38				
Income			0.96	0.42				
Social gather			−1.35	−0.22				
Social neighbor			−0.56	− 0.09				
Housing			0.38	0.25				
Discrimination			0.01	0.00				
Health Access			1.70	0.80				
Black					5.35**	2.81		
Other Race					−2.21*	−2.42		
Gender					0.18	0.07		
Employment					0.42	0.46		
Housing					−0.50	- 0.32		
Poverty					2.38*	1.00		
Higher Ed					−2.64*	−1.44		
HS Grad					−0.71	−0.61		
Literacy					1.60	0.41		
Health Access					2.19*	1.03		
Social Cohesion					−1.80	−0.28		
Social Cohesion- Neighbor					−0 .79	− 0.13		
Discrimination					1.24	0.09		
Arrest History					−2.23*	−0.99		
Community Safety					−0.37	−0.27		
Engaged Citizen-Vote					−0.11	−0.05		
Engaged Citizen-Volunteer					−0.62	−0.25		
Black							4.33**	2.38
Other Race							0.59	0.41
Gender							−0.30	−0.12
Employment							1.52	0.89
Poverty							0.60	0.25
Housing							0.65	0.46
Debt							2.46*	1.00
Literacy							1.37	1.23
HS Grad							0.12	0.10
Higher Ed							−2.74**	−1.54
Voc Training							0.36	0.25
Social Gather							−0.66	−0.11
Social Neighbor							0.03	0.05
Open Up-Spouse							0.98	0.87
Open Up-Children							−0.04	− 0.02
Open Up-Friend							1.29	0.94
Open Up-Family							−0.90	−0.54
Rely On-Spouse							−2.86**	−2.52
Rely On-Children							−0.06	−0.03
Rely On-Friend							−1.34	−0.91
Community-Vote							1.19	0.60
Community- Volunteer							0.59	0.22
Discrimination							−1.70	−0.14
Stress							−0.30	− 0.06
Housing							0.65	0.46
Neighborhood Safety							−0.87	−0.59
Health Access							−1.34	−0.79
Provider Availability							−0.35	−0.20
Healthcare Quality							12.25**	2.69
Political Context							1.66	0.72
**F Statistic**	7.76**		9.05**		6.04**		9.49**	
**R Square**	.06		.09		.07		.19	

*p < .05, **p < .01

**Table 4:** Logistic Regression for HbA1c

Independent Variable	County Health Ranking Model (1569)		WHO Model (1569)		Healthy People Model (1569)		Kaiser Model (1569)	
	OR	95% CI	OR	95% CI	OR	95% CI	OR	95% CI
Black	3.32**	2.30–4.80						
Other Race	2.32*	1.04–5.21						
Gender	1.29	0.90–1.83						
Housing	0.76	0.47–1.26						
Employment	1.13							
Education	0.91	0.50–1.66						
Rely On Spouse	0.93	0.49–1.05						
Rely On Children	0.88	0.62–1.25						
Rely On Friends	1.03	0.64–1.65						
Community Safety	1.10	0.64–1.87						
Income	1.59*	1.10–2.30						
Black			4.08**	2.74–6.06				
Other Race			2.28	0.96–5.43				
Gender			1.55*	1.08–2.23				
Political Context			0.97	0.64–1.48				
Social Class			0.91	0.83–1.02				
Education			0.91	0.49–1.63				
Work Status			1.12	0.68–1.85				
Income			1.33	0.90–1.98				
Social gather			1.12	0.97–1.29				
Social neighbor			0.99	0.85–1.16				
Housing			0.74	0.45–1.23				
Discrimination			0.98	0.92–1.04				
Health Access			1.31	0.87–1.93				
Black					3.84**	2.62–5.63		
Other Race					2.49*	1.11–5.62		
Gender					1.47*	1.02–2.14		
Employment					1.13	0.69–1.88		
Housing					0.64	0.40–1.04		
Poverty					1.56*	1.06–2.29		
Higher Ed					0.82	0.52–1.30		
HS Grad					0.94	0.46–1.89		
Literacy					2.92**	1.49–5.71		
Health Access					1.23	0.82–1.85		
Social Cohesion					1.13	0.98–1.30		
Social Cohesion-Neighbor					1.02	0.87–1.17		
Discrimination					1.00	0.94–1.07		
Arrest History					0.63*	0.42–0.96		
Community Safety					1.15	0.66–2.01		
Engaged Citizen-Vote					0.80	0.53–1.23		
Engaged Citizen-Volunteer					1.04	0.72–1.15		
Black							4.81**	3.02–7.65
Other Race							1.76	0.93–3.34
Gender							1.38	0.92–2.06
Employment							1.42	0.79–2.51
Poverty							1.43	0.93–2.19
Housing							0.83	0.46–1.48
Debt							0.78	0.53–1.16
Literacy							3.60**	1.74–7.27
HS Grad							0.95	0.44–2.02
Higher Ed							1.09	0.63–1.86
Voc Training							1.19	0.66–2.14
Social Gather							1.16	0.98–1.36
Social Neighbor							1.05	0.88–1.23
Open Up-Spouse							1.29	0.60–2.77
Open Up-Children							0.99	0.61–1.63
Open Up-Friend							0.72	0.37–1.45
Open Up-Family							0.96	0.53–1.71
Rely On-Spouse							0.58	0.27–1.25
Rely On-Children							0.91	0.54–1.52
Rely On-Friend							1.16	0.62–2.21
Community-Vote							0.97	0.60–1.57
Community- Volunteer							1.20	0.81–1.79
Discrimination							0.96	0.89–1.04
Stress							0.90	0.74–1.10
Housing							0.83	0.46–1.48
Neighborhood Safety							1.19	0.67–2.16
Health Access							1.24	0.72–2.11
Provider Availability							1.08	0.69–1.70
Healthcare Quality							1.81***	1.47–2.23
Political Context							1.06	0.67–1.66
**Wald χ2**	68.47**		72.85**		72.76**		107.36**	
**Naglekerke R** [2]	.09		.10		.09		.17	

*p < .05, **p < .01

## Discussion

The current study extends SDOH knowledge by empirically testing the four leading SDOH models (World Health Organization, Health Peoples Model, Kaiser Permanente, and the County Health Rankings Model) while analyzing their influence on diabetes risk factors. These finding elucidate the complex relationship between BMI, HbA1c, and social and environmental risk factors.

Each of the SDOH models indicated that Black individuals are at a higher risk for elevated HbA1c and BMI, compared to other racial/ethnic groups. Socioeconomic factors were also influential for predicting elevated HbA1c and higher BMIs. For example, poverty and literacy were associated with increased risk of both elevated HbA1c and higher BMIs. These findings align with existing research which indicate that Black individuals have increased risk for complications associated with diabetes [26] and that socioeconomic factors provide some explanation to these disparities [27]. Our study adds to this foundation of knowledge by examining the influence of race and socioeconomic factors in the context of SDOH models. Our research demonstrates that race, poverty, and literacy are highly predictive of elevated HbA1c and higher BMI even when accounting for other factors included in SDOH models.

Our study also provides valuable information regarding the collective influence of SDOH factors. There are several existing articles which examine SDOH factors such as poverty [16], race [28], and social support [9]. There are, however, fewer studies which examine these SDOH factors in relationship to one another. Further, our study is the first to our knowledge to examine SDOH models comprehensively. Our study, therefore, provides valuable information about how the factors included within SDOH models relate to one another. For example, economic status was included as a predictor within each SDOH model. Economic status was a significant predictor in most models for both elevated HbA1c and higher BMI but was nonsignificant in the Kaiser Model. This demonstrates that the influence of economic status is impacted by other SDOH factors. Future research may look at mediating and moderating relationships between economic factors and diabetes risk to better understand these relationships.

Last, there are no identified studies that analyze the influence of each SDOH for predicting elevated HbA1c and higher BMI. Our study fills this gap by providing information regarding how effective each SDOH model is for predicting elevated HbA1c and higher BMI. The Kaiser model was the most comprehensive and had the largest effect size for BMI even when using the adjusted R [2]. In addition, the Kaiser model had the largest effect for predicting elevated HbA1c when utilizing the Nagelkerke R [2].

### Kaiser model

The Kaiser model was most comprehensive including constructs such as social support, discrimination, and stress within their SDOH model. Individuals had higher BMIs when participants were Black, had debt, lacked access to higher education, did not have a spouse to rely on, and lacked access to quality healthcare. The Kaiser model was the only SDOH model to include variables of debt, stress, quality of care, and cultural competency of the healthcare provider. Framing SDOH in a comprehensive way increased the statistical impact of the Kaiser model regarding BMI even when using the adjusted R square.

### Healthy people model

The Healthy People model was the only one to include incarceration which was a significant predictor of HbA1c. In addition, the Healthy People model was more concrete in the way they operationalized their concepts. For example, they include poverty and housing instability rather than general categories of income and housing status. This supports the empirical construction of variables and therefore more readily allows for a standardized way to detect SDOH predictors of HbA1c.

### BMI and HbA1c

SDOH influencing elevated HbA1c include poverty, race, literacy issues, and lack of healthcare quality. Previous literature have examined the role of race and poverty on diabetes outcomes demonstrating racial minorities experienced higher risk of diabetes due to poverty and housing instability [16]. Our findings support those while adding additional context by testing housing and economic variables along with the other variables included within our statistical models. Regarding BMI, there were more main effects including social status, debt, education, and not having people to rely on. These findings suggest that there may be distinct risk factors for BMI and HbA1c. Social status, debt, lack of higher education, and lack of social support may influence HbA1c indirectly. Future research may examine the influence of these SDOH identifying whether these risk factors increase risk of developing diabetes over time.

### Intersections of poverty and race

Racial discrimination has been identified as a SDOH [29]. Much of the existing research evaluates the effect of racial discrimination on HbA1c [30], health behaviors [31], and BMI [32]. Few studies include other SDOH within their respective research models [33]. Our analysis demonstrated that Black individuals have increased risk for elevated HbA1c even when including several other SDOH variables. There were, in addition, significant findings for income, literacy challenges, and healthcare quality. These SDOH factors may be influential in how Black individuals experience increased risk. Research and policy practice may consider analyzing potential SDOH factors which are associated between racial identity and diabetes outcomes.

### Nonsignificant SDOH variables

Testing these models also demonstrated SDOH variables that were not statistically significant. For example, discrimination was included within two of the SDOH models, but was not significant for either BMI or HbA1c. In addition, social cohesion was included within 3 of the SDOH models but was only significant in the Health People Model for BMI. Some of these nonsignificant findings are contrary to existing research. For example, there are studies demonstrating the role of discrimination on diabetes outcomes [30], while other studies demonstrate a lack of significance when adding in additional control variables to include demographics and health behaviors [34]. There may be suppressor or mediating variables within the SDOH models. In addition, relying on subjective self-report data may be a limitation. Future research may build on existing research efforts to utilize more objective indicators of racial minority stress to include cortisol and C-reactive protein [35].

### Limitations

One limitation of the current study was that proxies were used to represent each SDOH construct, and that the Add Health dataset did not have a proxy for every construct included. Also, household income was reported, but the proportion of household income to the number of individuals within that household was not reported. The results regarding income should therefore be interpreted within the context of that limitation. Additionally, the dataset included a literacy variable, but did not specifically address health literacy. This study was cross sectional and, therefore, we cannot determine the longitudinal or causal relationship between the SDOH models and the health outcomes. Relying on self-report data may also be a limitation, and future research may utilize geospatial information system and social network analysis to better analyze environmental influences. Despite these limitations, this research provides a unique approach regarding methods to empirically test SDOH models.

### Future research

Longitudinal analyses may be performed to assess the influence of SDOH models and factors associated overtime. In addition, studies may include approaches to address social determinants of health (e.g., food as medicine programs) and identify how these interventions may impact diabetes outcomes. Future research may empirically test the association between the SDOH models and other disease states such as cardiovascular disease, chronic pain, and cancer. Since social workers are often utilized to address SDOH, randomized control trials (RCTs) may be employed which utilize social workers to assess SDOH and various healthcare concerns. Interventions to address these SDOH could be performed while assessing their impact on the SDOH indirectly and various healthcare outcomes indirectly. Additional areas of inquiry include 1) assessing the influence of healthcare providers’ perceptions of how SDOH influence diabetes-self management and how this may influence patient care interaction; 2) examining the association of psychiatric factors including depression and anxiety with SDOH factors; 3) exploring the impact of neighborhood factors such as social disorganization and discrimination; and 4) assessing the influence of SDOH risk on patient level perceptions of diabetes management and self-care.

## Conclusion

There is a need for health policies which equip healthcare providers and clinics to screen and address these SDOH. Specifically, there is a need for policies to promote value- based reimbursement incentives for healthcare systems that utilize universal screening to identify and address SDOH. Implementing policy that includes SDOH as an additional value-based incentive will motivate health care systems to screen for SDOH while providing the necessary funding for SDOH screening and intervention to be a sustainable practice. Neonatal screenings could also be provided for those with SDOH risk factors as a prevention strategy. Also screening for cardiovascular disease and other chronic illnesses within these populations is needed due to the high comorbidities of diabetes and cardiovascular risk [36]. There are existing screening tools such as FAMNEEDS [37], or the Health-Related Social Problems screener [38] that can be utilized to screen for SDOH. When considering which SDOH screener to use, those caring for diabetes should prioritize those which include variables that are statistically significant to include poverty, race and ethnicity, healthcare quality, social class, and education. In addition, policy makers need to emphasize legislation which equips health clinics to address SDOH influencing diabetes outcomes. Legislation should increase access to education, economic support for those in poverty, and increase access to quality health care. Increasing pay and incentives for providers to work in settings which serve those will less resources is also needed. Last, integrating clinical social workers, psychologists, and other behavioral health providers within traditional healthcare teams has demonstrated efficacy and is an additional strategy to mitigate the harmful effects of SDOH.

## Data Availability

The data that support the findings of this study are available from the corresponding author, ZC, upon reasonable request.
